# Analysis of Human Papillomavirus-HPV-18 and HPV-45 Type-specific variants in a contemporary cohort of individuals with cervical disease in Ghana

**DOI:** 10.4314/gmj.v58i4.9

**Published:** 2024-12

**Authors:** Gladys Kaba, Andrew Stevenson, Samuel A Sakyi, Thomas O Konney, Nicholas A Titiloye, Samuel A Oppong, Kwabena Amo-Antwi, Francis Agyemang-Yeboah, Kate Cuschieri, Sheila V Graham

**Affiliations:** 1 Department of Molecular Medicine, School of Medical Sciences, Kwame Nkrumah University of Science and Technology, Kumasi, Ghana, West Africa; 2 Department of Biomedical Sciences, School of Basic and Biomedical Sciences, University of Health and Allied Sciences, PMB 31 Ho, Volta Region, Ghana, West Africa; 3 Centre for Virus Research, School of Infection and Immunity, College of Medical, Veterinary and Life Sciences, Garscube Estate, University of Glasgow, Scotland, UK, G61 1QH; 4 Department of Obstetrics and Gynaecology, Komfo Anokye Teaching Hospital and Kwame Nkrumah University of Science and Technology, Kumasi, Ghana, West Africa; 5 Department of Pathology, School of Medical Sciences, Kwame Nkrumah University of Science and Technology, Kumasi, Ghana, West Africa; 6 Department of Obstetrics and Gynaecology, Korle-Bu Teaching Hospital and University of Ghana Medical School, Accra, Ghana, West Africa; 7 Scottish HPV Reference Laboratory, Department of Laboratory Medicine, Royal Infirmary of Edinburgh, University of Edinburgh, Scotland, UK, EH16 4SA

**Keywords:** Human papillomavirus, HPV-18/HPV-45 variants, lineage/sublineage

## Abstract

**Objectives:**

E6 and E7 DNA sequence **profile** of HPV-18 and HPV-45 lineage/sublineage variants in Ghana.

**Design:**

A cross-sectional study.

**Setting:**

Obstetrics/Gynaecology Directorate, Komfo Anokye Teaching Hospital, Kumasi and Department of Radiotherapy/Nuclear Medicine and the Family Planning Unit, Korle-Bu Teaching Hospital, Accra.

**Participants:**

207 individuals referred with clinical suspicion of cervical cancer (CxCa) or confirmed CxCa/precancer cases.

**Methods:**

Cervical swabs were collected (from October 2018 to November 2020) from individuals, with L1 DNA positivity for HPV-18(40 samples) and/or HPV-45(28 samples), out of 207 samples tested for 24-HPV-genotypes. DNA was extracted from a convenience sample and HPV-E6/E7-PCR (33/40-HPV-18-+ve- or 20/28-HPV-45-+ve samples), sequencing, and BLAST analysis was carried out.

**Results:**

After PCR amplification, the E6/E7 gene regions of 26 out of 33(HPV-18+ve) samples and **ten (10)** out of 20 (HPV-45+ve) samples were eligible for sequencing. For HPV-18 variants, 24 out of 26 samples (92.31%) were of lineage-B/C, including samples of lineage-C and 22 samples of lineage-B (out of which **ten (10)** samples were with E7-SNP-C665T). Nine out of ten HPV-45 variants were sublineage-A1, of which **two (2)** samples harboured both E6-SNPs-C134T and C4I7T including one sample with E6-SNPs-C134T, G415C, C4I7T detected together.

**Conclusions:**

Our study confirms a dominance of HPV-45-sublineage-A1 and HPV-18-lineage-B (with rare occurrence of Africa-specific HPV-18-lineage-C) variants in Ghana. Our study provides preliminary data on E6/E7 SNPs of HPV-18 and HPV-45 lineages and sublineages among CxCa cases in Ghana. We hope our data will inform future studies on pattern and distribution of HPV type-specific nucleotide changes that can be useful for therapeutic intervention.

**Funding:**

UK Government, (GK) Commonwealth Split-Site Scholarship with research support grant, number GHCN201823. GK also received Ghana public universities senior members book & research allowance, for research activities carried out in Ghana.

## Introduction

Human papillomavirus (HPV) is a common infection, and most individuals with sexual experience (current or past) are likely to become infected at some point. 1-3 Most HPV infections (~70 to 90%) are asymptomatic, transient and resolve within one to two years.^1^, ^2^ However, ~ 5% of individuals develop a persistent infection, which can lead to the progression of HPV-mediated cancer.^3^, ^4^

High-risk (HR) HPV types such as HPV-16, HPV-18 and HPV-45 infections are highly enriched (~74.4%) among CxCa cases compared with individuals without CxCa globally, including Africa. Among CxCa cases in West Africa compared to elsewhere, there is a higher percentage occurrence of HPV-45 (~16.2% versus ~5.0% ) and HPV-18 (~ 20.1% versus ~14.2% ) but a lower HPV-16 prevalence (~35.5% versus ~55.2%), though the combined prevalence of HPV-16/18/45^5^, ^6^ infection (~71.8%) is similar to the global estimate (~74.4%). HPV-18 and HPV-45 are classified in the alpha-7 species of HPVs, whilst HPV-16 belongs to the alpha-9 group.^7^

**A**n HPV genotype is defined as having L1 DNA sequence homology less than 10% from other classified types^7^, whereas variant lineages of a specific HPV type are classified by a nucleotide sequence difference of approximately 1.0% to 10.0% within the whole genome, while 0.5% to 1.0% is used to define sublineages. 8-10 Apart from HPV type (s), there is now evidence to suggest that specific lineages and sublineages of HPVs might influence infection persistence and/or clinical outcomes for HPV types such as HPV-16, 11-13 HPV-18^14^-^18^ and HPV-45.^19^, ^20^ The diversity of HPV type-specific variant lineages/sublineages is population-dependent. For HPV-18, there are three lineages (A/B/C) with eight sublineages (A1/A2/A3/A4/A5/B1/B2/B3) classified. 8, 15 For HPV-45, there are two lineages (A/B) with five classified sublineages (A1/A2/A3/B1/B2). Both B/C lineages of HPV-18 are predominant in sub-Sahara Africa 14 while HPV-45 sublineage-A1 is highly prevalent in the general African population.

Albeit, by whole genome sequencing, sequences within some specific HPV genome regions have delineated lineage and sublineage-specific and/or common SNP patterns for various HPV types, including HPV-18 & HPV-45. Incomplete HPV genome sequences can still be useful for classifying HPV-type specific lineages and sublineages in instances where whole genome sequencing may not be feasible (such as samples with insufficient quality/quantity DNA) and cost/technical implications, especially during epidemiological studies on natural history.^11^, ^19^, ^21^

The early (E) gene region of HPV contains the viral oncogenes E6 and E7. 22-25 From whole genome sequences of HPV-16, DNA polymorphisms in the E6 and E7 genes are fewer among CIN-3 and CxCa cases compared to individuals with lower grades of disease (<CIN3). Thus, increasing genomic variability of HPV-16 (especially within E7 DNA) was observed to be associated with decreasing cervical cancer risk. 13 There is a lack of data on genome conservation in the E6/E7 gene region in low-income countries, including Africa, with a higher burden of HPV-18 and HPV-45 infection. This study, therefore, sought to profile E6/E7 DNA sequence polymorphisms of type-specific variants, lineages, and sublineages of HPV-18 and HPV-45 using samples from a previously reported study population in Ghana. 26

## Methods

### Study participants and samples

This cross-sectional study included cervical swab samples from a clinical cohort of women referred to Komfo Anokye Teaching Hospital or Korle-Bu Teaching Hospital due to suspicion of cervical intraepithelial lesion and cancer as described in our previous report. 26 That is, individuals of the female gender with clinical suspicion of cancer of the cervix or confirmed diagnosis of CIN or CxCa referred for further medical assessment (including pelvic examination) and care, recruited from 9^th^ October 2018 to 10^th^ November 2020. DNA was extracted from the cervical swab samples using the QIAamp Media MDx DNA extraction Kit, and HPV genotyping was performed using the Optiplex HPV genotyping test (Diamex, Heidelberg, Germany) at the Scottish HPV Reference Laboratory (SHPVRL) in Edinburgh. All samples included in this current study were HPV-L1 DNA positive for HPV-18 (33 samples) and/or HPV-45 (20 samples), selected by convenience, serially, irrespective of histology. Subsequently, respective DNA extracts were sent to the Centre for Virus Research, University of Glasgow, Scotland, for HPV-18 or HPV-45 E6/E7 PCR, as detailed below.

### HPV-18 E6/E7PCR

HPV-18 DNA was amplified by PCR using ACCUZ-YME™ master mix (Bioline-UK); Accuzyme polymerase -high yield, high fidelity polymerase and following conditions stipulated by the manufacturer, as previously utilised by our group 26 with some modifications. The standard 25 µl PCR included 50 ng of DNA template; 0.2 µM HPV-18-E6-forward primer (FW)-5′CCG-AAA-ACG-GTC-GGG-ACC-G3′ and 0.2 µM HPV-18-E7-reverse primer (RV)-5′CGT-CTG-TAC-CTT-CTG-GAT-CAG3′ (Eurogentec-UK).

Primer design was based on the reported HPV-18 genome sequence (X05015.1) and was located 62 nucleotides before the E6 ATG start codon (from nucleotide 43 to 61 for HPV-18-E6 forward primer) and 31 nucleotides after the E7 stop codon (from nucleotides 938 to 918 for HPV-18-E7 reverse primer) with an expected E6/E7 PCR product size of 896 bp. PCR cycling conditions included an initial denaturation at 95.0 °C for 3 min, followed by 34 cycles of denaturation at 95.0 °C for 15 s, annealing at 60.0 °C for 5 s and extension at 72.0 °C for 2 min. The positive control was 50 ng DNA extract from HeLa cells (cervical carcinoma cell line with integrated HPV-18 DNA). To minimise the impact of low viral load/DNA concentration of HPV-18 or HPV-45 on PCR outcome, 50 ng of DNA template was utilised in an initial PCR, and where no product was observed, the reaction was repeated using 100 ng of DNA template. In all instances, a DNA template-free PCR negative control included nuclease-free water (Bioline-UK) instead of DNA. Subsequently, PCR products were separated on 1.5% agarose gels by electrophoresis and visualised under UV illumination.

### HPV-45 E6/E7 PCR

PCR analysis was carried out to amplify HPV-45 DNA using concentrations and cycling conditions, as described above, except for the primer set. HPV-45-E6 forward primer (FW)-5′GTG-TAA-CCG-AAA-ACG-GTT-GCA-ACC 3′ and HPV-45-E7 reverse primer (RV)-5′CCT-TCT-GGA-TCC-GCC-ATT-GTA-G3′ (Eurogentec-UK). HPV-45 E6/E7 primers were designed based on the reported HPV-45 genome (X74479.1). The forward primer was located 68 nucleotides before the E6 ATG start codon (from nucleotide 34 to 57 for HPV-45-E6-FW) and 23 nucleotides after the E7 stop codon (from nt 930 to 909 for HPV-45-E7-RV) giving an expected E6/E7 PCR product of 899 bp.

### DNA sequencing

PCR products from samples with the expected E6/E7 DNA band size were purified and sequenced using Sanger sequencing (Eurofins-UK) with forward and reverse sequencing primer replicate reactions. HPV-18-E6/E7-sequencing primer set; HPV-18-E6-FW-5′CGG-TCG-GGA-CCG-AAA-ACG-GTG3′ (from nucleotide 50 to 70) & HPV-18-E7-RV-5′CCT-TCT-GGA-TCA-GCC-ATT-GTT-GC3′ (from nucleotide 930 to 908) with expected DNA size of 881 bp.

HPV-45-E6/E7-sequencing primer set; HPV-45-E6-FW-5′CGG-TTG-CAA-CCA-AAA-ACG-GTG-C3′ (from nucleotide 47 to 68) & HPV-45-E7-RV-5′GGA-TCC-GCC-ATT-GTA-GAT-TAT-TGG 3′ (from nucleotide 924 to 901) with expected DNA size of 878 bp. Two sequencing reactions were done independently on each sample. Nucleotide changes were reported only if the same changes were discovered using both forward and reverse sequencing-primers sequences in both replicates.

### Analysis of HPV-18 or HPV-45 E6/E7 DNA sequences

BLAST (https://blast.ncbi.nlm.nih.gov/Blast.cgi), was used to analysed HPV-18 (& HPV-45) E6/E7 DNA sequences. Human papillomavirus variants were classified according to reported lineage and sublineage-specific, and common E6/E7 SNP patterns of HPV-18 8, 27 (& HPV-45 8, 19 ) and summarised together with previously reported HPV-18 ( & HPV-45) variant genomes. 15, 28, 29 Supplementary Figures 1 and 2 show annotated HPV-18 and HPV-45 E6/E7 homologies to previously reported respective reference genomes and sublineages, indicating lineage and sublineage-related SNP patterns.

**Supplementary-Figure-1:**
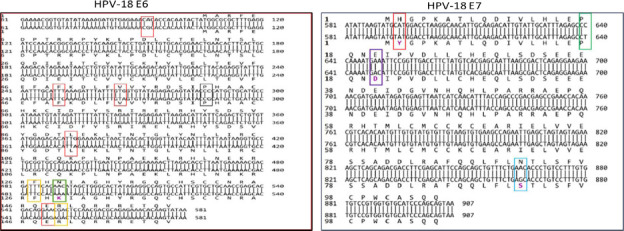
An annotated HPV-18-E6/E7 nucleotide - nucleotide BLAST of sublineage-A1 (X05015.1 - reference genome; upper strand) and sublineage-B2 (KC470225.1; lower strand) **E6**: **Red box** - common SNPs (T251C / G266A) of lineage-B/C & Lineage-specific SNPs (G374A / A548G) of lineage- B/C ; **Black box** - C287G- among SNPs initially reported in HPV-18-reference (X05015.1)^28^ but were subsequently updated; 29 **Yellow box** - non -lineage-specific SNPs (T485C / C549A) of A2 / A3 / A4 / A5 & B/C; **Green box** - non-lineage SNP C491A (amino acid residue change N129K) but reported in most detected lineage-B variants. 8, 27, 34 **E7**: **Red box** - lineage-specific SNP C593T (amino acid residue change H2Y) of lineage-B/C; **Green box** - lineagespecific SNP (C640T) of lineage-B; **Purple box** - non-lineage-specific SNP A649C (amino acid residue change E20D) reported among some sublineage-B2; **Blue box**- lineage- specific SNP A864G (amino acid residue change N92S) of both B1/B2. 8, 34 NB- Aspartic acid = D; Glutamic acid = E; Histidine = H; Lysin = K; Serin = S; Tyrosine = Y

**Supplementary-Figure-2:**
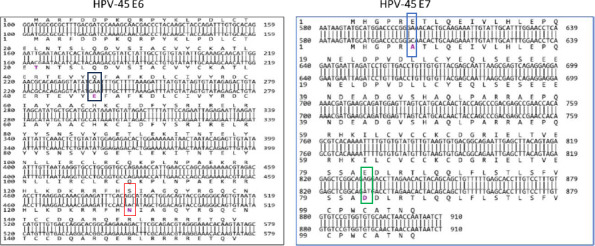
An annotated HPV-45-E6/E7 nucleotide - nucleotide BLAST of sublineage-A1 [(X74479- reference genome; upper strand) and sublineage-A2 (EF202157; lower strand) **E6: Black box** - non-lineage-specific SNP C237G (amino acid residue change Q46E); **Red box** - sublineage-specific SNP G487A of sublineage-A2 **E7: Blue box** - non-lineage-specific SNP A603C (amino acid residue change E6A); **Green box** - non-lineage-specific SNP G832T (amino acid residue change E82D) NB- Alanine = A; Aspartic acid =D; Glutamic acid=E; Q = Glutamine

### Ethical clearance

Ethical review and clearance was attained from the Committee on Human Research Publication and Ethics, School of Medical Sciences, Kwame Nkrumah University of Science and Technology/Komfo Anokye Teaching Hospital (CHRPE/AP/559/18) and Scientific Technical Committee/Institutional Review Board, Korle-Bu Teaching Hospital (KBTH-IRB/0081/2018). Inform consent was obtained from study participants before cervical swab sampling was carried out during routine clinical care, as detailed in Kaba *et al.*, 2023.^26^

## Results

### Demographic and clinical characteristics and HPV profile

In our previous report 26, LBC samples from 207 individuals referred with clinical suspicion of cervical cancer (CxCa) or confirmed CxCa/precancer cases samples were analysed by HPV L1 genotyping. Among these, 68 out of 207 samples were positive for HPV-18 (40 samples) and/or HPV-45 (28 samples). Details on frequency distribution of age group, histology, and HPV profile among individuals with HPV-18-L1+ and HPV-45-L1+ samples are summarised respectively in Table 1 and Table 2.

**Table 1:** Frequency Distribution of Age Group, Histology, And HPV Profile of Samples HPV-18 positive

HISTOLOGY		AGE GROUP (YEARS)				HPV PROFILE	
		20-39	40-59	60-79	≥80	1HPV	>1HPV
	N (%)	N	N	N	N	N	N
**Squamous Cell Carcinoma**	30 (70.0)	2	13	11	4	16	14
**Adenocarcinoma**	4 (10.0)	1	2	0	1	2	2
**Adenocarcinoma in Situ (AIS)**	1 (2.5)	0	1	0	0	0	1
**CxCa (unconfirmed morphology)**	2 (5.0)	0	1	1	0	1	1
**Histology not done**	1 (2.5)	0	0	1	0	1	0
**CIN-3**	1 (2.5)	0	0	1	0	1	0
**NILM**	1 (2.5)	1	0	0	0	0	1
**TOTAL**	40 (100.0)	4	17	14	5	21	19

*CxCa=Cervical Cancer, NILM= Negative for Intraepithelial Lesion or Malignancy, CIN-3= Cervical Intraepithelial Neoplasm Grade 3*

**Table 2:** Frequency Distribution of Age Group, Histology, and HPV Profile of Samples HPV-45 positive

HISTOLOGY		AGE GROUP (YEARS)				HPV PROFILE	
		20-39	40-59	60-79	≥80	1HPV	>1HPV
	N (%)	N	N	N	N	N	N
**Squamous Cell Carcinoma**	19 (67.9)	2	6	8	3	10	9
**Adenocarcinoma**	2 (7.1)	0	0	1	1	2	0
**Chronic Inflammation – Cervical**	1 (3.6)	0	1	0	0	1	0
**ASCUS**	1 (3.6)	1	0	0	0	1	0
**CIN3**	1 (3.6)	0	1	0	0	0	1
**NILM- Previous HPV Positive**	1 (3.6)	1	0	0	0	0	1
**Vaginal Cancer (Post CxCa)**	1 (3.6)	0	0	1	0	1	0
**Undetermined Histology**	2 (7.1)	0	0	1	1	1	1
**TOTAL**	28 (100.0)	4	8	11	5	16	12

*CxCa = Cervical Cancer, NILM = Negative for Intraepithelial Lesion or Malignancy, CIN-3 = Cervical Intraepithelial Neoplasm Grade , ASCUS = Atypical Squamous Cells of Undetermined Significance*

After the day 1 interview, participants were asked if they had any concerns they needed answers for. One hundred and fifty-seven participants (70%) wanted to know when they would receive their biopsy reports, where to go for the report and what to do with it. They also admitted to being anxious about the outcome of their biopsy. One hundred and sixty-eight participants (75%) out of the 224 participants interviewed on the first day after the procedure showed gratitude to the research assistant for calling to follow up on them.

This study detected two out of 26 samples of HPV-18-lineage-A, out of which one sample had an E6/E7 nucleotide sequence that was synonymous with sublineage-A2/A3/A4 whilst another sample with nucleotide changes -A92G/T104C/A377G/C547A/C665T was likely of sublineage-A5. Additionally, 24 out of 26 samples (88.5%) were of HPV-18-B/C-lineages, including 22 samples of lineage-B and 2 samples of lineage-C. Within the HPV-18-B-lineage, sublineages included either B1 and/or B2 (ten samples), B3 (nine samples) and one sample of B4-sublineage. Ten out of 22 samples of the HPV-18-B-lineage contained E7 SNP (C665T), including seven samples (sublineage -B1 and/or B2) and one sample of sublineage-B3. An additional two samples of HPV-18-lineage-B showed E7- SNP-C665T but could not be classified into sublineage (s). One sample had two possible nucleotide changes (at A864G/A), while another lacked discriminatory information at E7 nucleotides 864 and 865. The only sample of HPV-18-sublineage-A5 classified in this study also harboured E7 SNP (C665T) (Supplementary Table 1). Apart from naturally occurring lineage/sublineage-specific and common SNPs of HPV-18 E6/E7 DNA sequences outlined above, we detected other SNPs in ten out of 26 samples, and these are detailed in Supplementary Table 2.

**Supplementary-Table-1:** Polymorphisms of HPV-18 E6/E7 DNA Sequence, among 26 Individuals, Summarized Alongside Previously Reported HPV-18 Lineage and Sublineage Variant Genomes

**HPV-18 Variant**	**92**	**104**	**106IN107**	**149**	**232**	**251**	**266**	**287**	**317**	**342**	**374**	**377**	**437**	**485**	**491**	**500**	**501**	**548**	**549**	**551**	**593**	**640**	**649**	**664**	**665**	**736**	**751**	**864**	**865**	**874**
**X05015.1 (HPV-18-Reference)**	**A**	**T**		**T**	**A**	**T**	**G**	**C**	**T**	**C**	**G**	**A**	**G**	**T**	**C**	**G**	**A**	**A**	**C**	**A**	**C**	**C**	**A**	**T**	**C**	**A**	**C**	**A**	**C**	**C**
**A1 (AY262282 .1)**	**_**	**_**		**_**	**_**	**_**	**_**	**G**	**_**	**_**	**_**	**_**	**_**	**_**	**_**	**_**	**_**	**_**	**_**	**_**	**_**	**_**	**_**	**_**	**_**	**_**	**_**	**_**	**_**	**_**
**A2 (EF202146.1)**	**_**	**_**		**_**	**_**	**_**	**_**	**G**	**_**	**_**	**_**	**_**	**_**	**C**	**_**	**_**	**_**	**_**	**A**	**_**	**_**	**_**	**_**	**_**	**_**	**_**	**_**	**_**	**_**	**_**
**A3 (EF202147.1)**	**_**	**C**		**_**	**G**	**_**	**_**	**G**	**_**	**_**	**_**	**_**	**_**	**C**	**_**	**_**	**_**	**_**	**A**	**_**	**_**	**_**	**_**	**_**	**_**	**_**	**T**	**_**	**_**	**T**
**A4 (EF202151.1)**	**_**	**C**		**_**	**_**	**_**	**_**	**G**	**_**	**_**	**_**	**_**	**_**	**C**	**_**	**_**	**_**	**_**	**A**	**_**	**_**	**_**	**_**	**C**	**_**	**_**	**T**	**_**	**_**	**_**
**A5 (GQ180787.1)**	**G**	**C**		**C**	**_**	**_**	**_**	**G**	**_**	**_**	**_**	**G**	**_**	**C**	**_**	**_**	**_**	**_**	**A**	**_**	**_**	**_**	**_**	**_**	**_**	**_**	**_**	**_**	**_**	**_**
**B1 (EF202155.1)**	**G**	**_**		**_**	**_**	**C**	**A**	**G**	**C**	**T**	**A**	**_**	**_**	**C**	**A**	**_**	**_**	**G**	**A**	**G**	**T**	**T**	**_**	**_**	**_**	**G**	**_**	**G**	**_**	**_**
**B2 (KC470225.1)**	**G**	**_**		**_**	**_**	**C**	**A**	**G**	**_**	**_**	**A**	**_**	**_**	**C**	**A**	**_**	**_**	**G**	**A**	**_**	**T**	**T**	**C**	**_**	**_**	**_**	**_**	**G**	**_**	**_**
**B3 (EF202152.1)**	**G**	**_**		**_**	**_**	**C**	**A**	**G**	**_**	**_**	**A**	**_**	**_**	**C**	**A**	**_**	**_**	**G**	**A**	**_**	**T**	**T**	**_**	**_**	**_**	**_**	**_**	**_**	**A**	**_**
**C (KC470229.1)**	**G**	**_**		**_**	**_**	**C**	**A**	**G**	**_**	**_**	**A**	**_**	**A**	**C**	**_**	**_**	**_**	**G**	**A**	**_**	**T**	**_**	**_**	**_**	**_**	**_**	**_**	**_**	**_**	**_**
																														
**Samples (Variant/Histology)**																														
**A/A5 (1xSCC)**	**G**	**C**		**_**	**_**	**_**	**_**	**G**	**_**	**_**	**_**	**G**	**_**	**C**	**_**	**_**	**_**	**_**	**A**	**_**	**_**	**_**	**_**	**_**	**T**	**_**	**_**	**_**	**_**	**_**
**A (1xCervical Cancer)**	**_**	**_**	**G/_**	**_**	**_**	**_**	**_**	**G**	**_**	**_**	**_**	**_**	**_**	**C**	**_**	**_**	**_**	**_**	**A**	**_**	**_**	**_**	**_**	**_**	**_**	**_**	**_**	**_**	**_**	**_**
**C (2xSCC)**	**G**	**_**		**G/-**	**_**	**C**	**A**	**G**	**_**	**_**	**A**	**_**	**A**	**C**	**_**	**_**	**_**	**G**	**A**	**_**	**T**	**_**	**_**	**_**	**_**	**_**	**_**	**_**	**_**	**_**
**B (1xSCC)**	*	**_**	**G**	**_**	**_**	**C**	**A**	**G**	**_**	**_**	**A**	**_**	**_**	**_**	**A**	**_**	**_**	**G**	**A**	**_**	**T**	**T**	**_**	**_**	**T**	**_**	**_**	**_/G**	**_**	**_**
**B (1xSCC)**	*	*		*	**_**	**C**	**A**	**G**	**_**	**_**	**A**	**_**	**_**	**C**	**A**	**_**	**_**	**G**	**A**	**_**	**T**	**T**	**_**	**_**	**T**	**_**	**_**	*	*	*
**B1/B2 (1xSCC)**	**G**	**_**		**_**	**_**	**_**	**A**	**G**	**_**	**_**	**A**	**_**	**_**	**_**	**A**	**_**	**_**	**G**	**A**	**_**	**T**	**T**	**_**	**_**	**T**	**_**	**_**	**G**	**_**	**_**
**B1/B2(1xADC; 1xNILM)**	**G**	**_**		**_**	**_**	**C**	**A**	**G**	**_**	**_**	**A**	**_**	**_**	**C**	**A**	**_**	**_**	**G**	**A**	**_**	**T**	**T**	**_**	**_**	**_**	**_**	**_**	**G**	**_**	**_**
**B1/B2 (5xSCC; 1xADC)**	**G**	**_**		**_**	**_**	**C**	**A**	**G**	**_**	**_**	**A**	**_**	**_**	**C**	**A**	**_**	**_**	**G**	**A**	**_**	**T**	**T**	**_**	**_**	**T**	**_**	**_**	**G**	**_**	**_**
**B1/B2 (1xSCC)**	**G**	**_**	**G/_**	**_**	**_**	**C**	**A**	**G**	**_**	**_**	**A**	**_**	**_**	**C**	**A**	**_**	**_**	**G**	**A**	**_**	**T**	**T**	**_**	**_**	**_**	**_**	**_**	**G**	**_**	**_**
**B3 (6xSCC)**	**G**	**_**		**_**	**_**	**C**	**A**	**G**	**_**	**_**	**A**	**_**	**_**	**C**	**A**	**_**	**_**	**G**	**A**	**_**	**T**	**T**	**_**	**_**	**_**	**_**	**_**	**_**	**A**	**_**
**B3 (1xSCC)**	**G**	**_**	**G**	**_**	**_**	**C**	**A**	**G**	**_**	**_**	**A**	**_**	**_**	**C**	**A**	**_**	**_**	**G**	**A**	**_**	**T**	**T**	**_**	**_**	**_**	**_**	**_**	**_**	**A**	**_**
**B3 (1xADC)**	*	*		**_**	**_**	**C**	**A**	**G**	**_**	**_**	**A**	**_**	**_**	**C**	**A**	**_**	**_**	**G**	**A**	**_**	**T**	**T**	**_**	**_**	**T**	**_**	**_**	**_**	**A**	**_**
**B3 (1xSCC)**	*	**_**		**_**	**_**	**C**	**A**	**G**	**_**	**_**	**A**	**_**	**_**	**C**	**A**	**_**	**_**	**G**	**A**	**_**	**T**	**T**	**_**	**_**	**_**	**_**	**_**	**_**	**A**	**_**
**B4 (1xSCC)**	**G**	**_**	**G/_**	**_**	**_**	**C**	**A**	**G**	**C**	**_**	**A**	**_**	**_**	**C**	**A**	**A**	**T**	**G**	**A**	**_**	**T**	**T**	**_**	**_**	**_**	**_**	**_**	**G**	**_**	**_**

The table shows in rows 2-11 reference genomes for HPV-18 wild type (row 2) and variant genomes. Rows 1326 show the 26 HPV-18-positive samples with E6/E7 DNA sequences, out of which 24 samples were of HPV-18 lineages-B/C. In each column, the number in the first row represent a single nucleotide position within the HPV-18-E6/E7 DNA.

HPV-18-B/C-specific SNPs / common SNPs = G374A/A548G/T251C/G266A/G374A/A548G & C593T

HPV-18-lineage-C-specific SNP = G437A

HPV-18-lineage- B-specific SNPs = C640T/C491A

HPV-18-sublinaege-B1/B2-Specific SNP = A864G

HPV-18-sublineage-B3-specific SNP = C856A;

HPV-18-sublineage-B4 candidate - specific SNPs = G500A/C501T

NB: - = same as the reference genome; * = nucleotide base data not available; NILM = Negative for Intraepithelial Lesion or Malignancy; SCC = Squamous cell carcinoma; ADC=Adenocarcinoma

**Supplementary-Table 2:** Human Papillomavirus type -18 E6/E7 SNPs that occurred in a lone sample

Nucleotide Changes	Reference
T245C	
G495 G/A*	
G280	G280A, leading to amino acid change S59N 14
C460C/G*; T787T/G*	
G500A; C501T	C501T, leading to amino acid change H133Y 14, 18
A770C	18, 42
C158 C/T*; T664G	But T664C 8
A218T	14, 18
C148 C/G*; C822C/T*	
T149G	T149C/G 14

NB-* = dual nucleotide changes at one nucleotide position; Bolded SNPs have been p

### HPV-45 E6/E7 DNA sequence polymorphisms

Out of 28 (HPV-45-+ve samples), 20 were analysed by E6/E7 PCR. Eleven gave the expected HPV-45 E6/E7 DNA band at 899 bp, out of which ten samples (including seven CxCa cases, one sample from a CIN-3 case, another one sample from a case with suspicion of recurrence of cervical cancer after treatment/management and another sample where histology was not available) yielded adequate sequences for analysis- one sample with PCR product was not sent for sequencing because of an oversight. E6/E7 PCR could not be carried out on 8/28 HPV45-positive samples as HPV genotyping had not been carried out on these samples, whilst nine samples gave no E6/E7 PCR product though repeated by doubling the DNA template concentration. Table 3 summarises sublineage-specific and common E6/E7 SNPs of HPV-45. Briefly, only one sample was observed with sublineage-A2-specific SNP (4G487A), and nine out of ten samples (90.0%) were of HPV-45-sublineage-A1, all with the nucleotide change G600A. Two of these nine samples (of HPV-45-sublineage-A1) had both E6-SNPs C134T and C4I7T, including one sample harbouring an additional E6-SNP (G415C). Supplementary Table 3 summarises observed HPV-45 E6/E7 SNPs among ten individuals with respective histology.

**Table 3:** E6 and E7 Single nucleotide polymorphisms (SNPs) of HPV-18 and HPV-45 variants

SNP(s)	Lineage	N (%)	SNP(s)	Sublineage	N (%)
HPV-18 (N = 26)					
T251; G266; G374; A548; C593	A	2 (7.7)	A92G; T104C; A377G; C547A; C665T	A5	1(3.8)
T251C; G266A; ^a^G374A;^b^C491A; ^a^A548G; ^a^C593T; C640T	B	22 (84.6)	^a^A864G	B1 / B2	10 (38.5)
			^a^C865A	B3	9 (34.6)
			^a^G500A ; ^a^C501T	B4	1 (3.8)
**T251C; G266A; ^a^G374A; G437A; ^a^A548G; ^a^C593T**	C	2 (7.7)			
**HPV-45 (N = 10)**					
	A	10 (100.0)	G600	A1	9 (90.0)
			^a^G487A	A2	1(10.0)

a Reported HPV-18-lineage/sublineage or HPV-45-sublineage specific SNP (s)

b Nucleotide change at C491A of HPV-18 E6 DNA, has been reported with an increased concentration of E6*I transcript in lineage-B than Lineage-A though outside donor / acceptor splice site. 30-32

**Supplementary-Table-3:** Polymorphisms observed within HPV-45 E6/E7 DNA sequence among ten individuals, summarised alongside previously reported HPV-45 lineage and sublineage variant genomes

**HPV-45 Variant**	**124**	**134**	**146**	**150**	**162**	**163**	**237**	**259**	**277**	**284**	**286**	**295**	**350**	**382**	**400**	**415**	**417**	**482**	**487**	**497**	**529**	**590**	**600**	**603**	**649**	**650**	**694**	**718**	**769**	**772**	**795**	**808**	**832**	**836**
**A1(X74479)**	**A**	**C**	**A**	**T**	**T**	**T**	**C**	**G**	**T**	**A**	**A**	**G**	**C**	**C**	**A**	**G**	**C**	**T**	**G**	**A**	**A**	**C**	**G**	**A**	**A**	**G**	**G**	**A**	**A**	**T**	**A**	**G**	**G**	**C**
**A2(EF202157)**	**_**	**_**	**_**	**_**	**A**	**C**	**G**	**_**	**_**	**_**	**_**	**_**	**_**	**_**	**_**	**_**	**_**	**C**	**A**	**_**	**_**	**_**	**_**	**C**	**_**	**_**	**_**	**_**	**_**	**_**	**_**	**_**	**T**	**_**
**A3(KC470256)**	**_**	**_**	**G**	**C**	**_**	**_**	**_**	**_**	**_**	**_**	**_**	**_**	**_**	**_**	**_**	**_**	**_**	**C**	**_**	**G**	**_**	**T**	**A**	**C**	**G**	**_**	**_**	**_**	**C**	**_**	**_**	**_**	**T**	**T**
**B1(EF202161)**	**_**	**_**	**_**	**_**	**_**	**_**	**_**	**_**	**_**	**C**	**_**	**_**	**_**	**_**	**_**	**_**	**_**	**C**	**_**	**G**	**_**	**_**	**_**	**C**	**_**	**_**	**_**	**C**	**_**	**_**	**_**	**T**	**T**	**_**
**B2 (EF202164)**	**C**	**_**	**_**	**_**	**_**	**_**	**_**	**T**	**_**	**_**	**_**	**_**	**_**	**_**	**_**	**_**	**_**	**C**	**_**	**G**	**_**	**_**	**A**	**C**	**_**	**_**	**_**	**C**	**_**	**_**	**_**	**_**	**T**	**_**
**Samples (Variant/Histogy)**																																		
**A2/1xSCC**	**_**	**_**	**_**	**_**	**_**	**_**	**_**	**_**	**_**	**_**	**_**	**_**	**_**	**_**	**_**	**_**	**_**	**C**	**A**	**_**	**_**	**_**	**_**	**C**	**_**	**_**	**_**	**_**	**_**	**_**	**T**	**_**	**T**	**_**
**A1/1xVaginal Cancer (Post CxCa)**	**_**	**_**	**_**	**_**	**_**	**_**	**_**	**_**	**_**	**_**	**_**	**_**	**_**	**_**	**_**	**_**	**_**	**_**	**_**	**_**	**_**	**_**	**_**	**_**	**_**	**_**	**_**	*	*	*	*	*	*	*
**A1/1xUKN Histology**	*	*	*	*	*	*	*	**_**	**_**	**_**	**_**	**_**	**_**	**_**	**_**	**_**	**_**	**_**	**_**	**_**	**_**	**_**	**_**	**_**	**_**	**_**	*	*	*	*	*	*	*	*
**A1/1xCIN3**	**_**	**_**	**_**	**_**	**_**	**_**	**_**	**_**	**_**	**_**	**_**	**_**	**_**	**_**	**_**	**_**	**_**	**_**	**_**	**_**	**_**	**_**	**_**	**_**	**_**	**_**	**_**	**_**	**_**	**_**	**_**	**_**	**_**	**_**
**A1/1xADC**	**^_^**	**T/-**	**_**	**_**	**_**	**_**	**_**	**_**	**_**	**_**	**_**	**_**	**_**	**_T/**	**_T**	**_/C**	**_/T**	**_**	**_**	**_**	**_**	**_**	**_**	**_**	**_**	**_**	**_**	**_**	**_**	**_**	**_**	**_**	**_**	**_**
**A1/1xSCC**	**^_^**	**T/-**	**_**	**_**	**_**	**_**	**_**	**_**	**_**	**_**	**T/-**	**_**	**_**	**_**	**_**	**_**	**T/_**	**_**	**_**	**_**	**_**	**_**	**_**	**_**	**_**	**_**	**_**	**_**	**_**	**_**	**_**	**_**	**_**	**_**
**A1/1xSCC**	**_**	**_**	**_**	**_**	**_**	**_**	**_**	**_**	**_/G**	**_**	**_**	**_**	**_/T**	**_**	**_**	**_**	**_**	**_**	**_**	**_**	**_**	**_**	**_**	**_**	**_**	**_**	**A/-**	**_**	**_**	**_**	**_**	**_**	**_**	**_**
**A1/1xSCC**	**_**	**_**	**_**	**_**	**_**	**_**	**_**	**_**	**_**	**_**	**_**	**C/_**	**_**	**_**	**_**	**_**	**_**	**_**	**_**	**_**	**_**	**_**	**_**	**_**	**_**	**_/A**	**_**	**_**	**_**	**_**	**_**	**_**	**_**	**_**
**A1/1xSCC**	*	*	*	*	*	*	**_**	**_**	**_**	**_**	**_**	**_**	**_**	**_**	**_**	**_**	**_**	**_**	**_**	**_**	**_**	**_**	**_**	**_**	**_**	**_**	**_**	**_**	**_**	C/*	**_**	**_**	**_**	**_**
**A1/1xSCC**	**_**	**_**	**_**	**_**	**_**	**_**	**_**	**_**	**_**	**_**	**_**	**_**	**_**	**_**	**_**	**_**	**_**	**_**	*	*	**C**	**_**	**_**	**_**	**_**	**_**	**_**	**_**	**_**	**_**	**_**	**_**	**_**	**_**

The table shows in rows 2-6 reference genomes for HPV-45 wild type (row 2) and variant genomes. Rows 8-17 show the ten HPV-45-positive samples with E6/E7 DNA sequences, out of which nine samples were of HPV-45-sublineages-A1. In each column, the number in the first row represent a single nucleotide position within the HPV-45-E6/E7 DNA. HPV-45 E7 SNP G600 was detected in all sublinege-A1 detected in this study, NB -HPV-45 E7 SNP G600 though also detected among sublineages-A3/B2 , however, the reported specific SNPs for -A3; -B2; -A3/B1/B2 19 , detailed below, was not detected in this study

HPV-45-sublineage-A2-specific SNP = G487A

HPV-45-sublineage-A3-specific SNPs = A146G / T150C

HPV-45-sublineage-B2-specific SNP = A124C

HPV-45-sublineages-A3/B1/B2-specific SNP = A497G

NB: - = same as the reference genome; *= nucleotide base data not available; CxCa = cervical cancer; SCC-squamous cell carcinoma; ADC=Adenocarcinoma;

## Discussion

This study analysed E6/E7 DNA sequences from 26 HPV-18 +ve samples and ten HPV-45 positive samples to determine type-specific lineages/sublineages. Among HPV-18 variants detected in this cohort, only two samples were lineage-A, while 24 out of 26 samples (92.3%) were lineages-B/C (22 samples of lineage-B and 2 samples of lineage-C), similar to that reported previously in Sub-Saharan Africa. 8, 14, 33 Rare occurrences of HPV-18-lineage-C (though specific to Africa) have been reported in Rwanda, Burkina Faso, and Zambia.^8^ per our study, and Benin, Guinea, Kenya, Mali, Nigeria, Rwanda, South Africa, Tanzania, and Uganda.^14^ Additionally, a previous study in Ghana using cervical swab samples collected during a community-based cervical cancer screening from women with normal cytology also reported a high percentage occurrence of HPV-18-lineage-B (9 out of 12 samples, 83.33%) compared with lineage-C (1 out of 12 samples).^33^ However, a predominance of HPV-18-lineage-A has been reported in most regions across the globe, including North Africa-Morocco and Algeria ( with over 90% occurrence) but with the exception of Sub-Saharan Africa.^14^

Almost all of our classified HPV-18-lineage-B/C variants harboured previously reported lineages-B/C-common E6 SNPs (T251C/G266A) and lineages-B/C-specific SNPs within E6 (G374A/A548G) and E7 (C593T), 8 except one sample of sublineage-B1/B2 without the T251C nucleotide change. Instead, nucleotide 251 was unchanged compared to the HPV-18 reference genome (X05015.1), and this has been observed before among some HPV-18-lineage-C variants. 14 However, we report for the first time in Ghana that E6 SNPs G500A and C501T, specific for HPV-18-candidate-sublineage-B4, first described among six out of 708 exfoliated cells or tissue biopsy samples from Kenya, Rwanda, Cuba, and Brazil among thirty-nine countries worldwide. 14

All detected HPV-18-lineage-B variants in our study were with E6 SNP C491A (with the amino acid change N129K) that was previously reported to be specific for HPV-18 lineage-B 34 but subsequently, was not detected in some sublineage-B2 variants^8^ whilst a fraction of sublineage-B3 has been detected with another nucleotide change (C491T). 14 However, E6 SNP C491A does not affect E6 isoform expression, but a higher concentration of E6*I transcript among HPV-18-lineage-B samples (with this nucleotide change—C491A) compared with lineage-A has been reported.^30^-^32^

Our current study observed a higher percentage occurrence of the E7 C665T SNP among either B1 and/or B2 sublineage groups (70.0%, seven out of ten samples) compared with other HPV-18- sublineages. The nucleotide change, E7 SNP C665T, has been reported with an unchanged amino acid at residue 26 of the E7 protein important for carcinogenesis.^8^ because the pRB binding site is from amino acid residues 25 to 29. 35 Though not sublineage-specific, the E7 nucleotide change C665T was previously reported in some sublineage-B2 isolates but not sublineage-B1.^8^ However, further investigation into the linkages of E7 SNP C665T to other nucleotide changes in other region(s) within the HPV-18 genome and/or into how such non-lineage/sublineage-specific SNPs were created may be needed.

In this study, we detected either HPV-18- B1 and/or B2 sublineage(s) together among ten out of twenty-two samples of lineage-B but could not distinguish between sublineage-B1 and B2 because there are no specific SNP (s) for B1 or B2 within E6/E7 DNA sequence. 8 Albeit, Chen et al., 2013, reported HPV-18-sublineage-B1 to be predominant in Southern-Central Africa-Rwanda (60%, 16 out of 26 samples) and Zambia (50%, six out of twelve samples ) whilst in Burkina Faso (Sub-Sahara Africa, a neighbour to Ghana) there were almost equally prevalent sublineages B2 (46.0%, five out of eleven samples) and B3 (36.4%, four out of eleven samples). 8

Additionally, a study in Ghana by LCR DNA sequencing among individuals without cervical cancer detected HPV-18-sublineage-B2 (50.0%, six out of twelve samples) in the majority compared to sublineage-B1 (in one sample) and sublineage-B3 (two out of twelve samples).^33^

Similar to other studies in Africa, including a previous study in Ghana among individuals without cancer of the cervix, 33 this study detected HPV-45-sublineage-A1 in the majority (9/10, 90.0%) of cases. A dominance of HPV-45-sublineage-A1 has been reported in Africa at 63.2%: Guinea, Kenya, Mali, Morocco, Nigeria, Senegal, South Africa, Tanzania, and Uganda.^19^ Both HPV-45 Sublineages B1 and B2 have been reported in most regions worldwide, including 22.6% in Africa.^19^

We also detected E6-SNPs C134T & C4I7T together in two samples of HPV-45-sublineage-A1, including one sample with an additional E6-SNPs-G415. The nucleotide change C134T has been previously reported among sublineage-B2, 19 but the C4I7T change has not been reported 8, 19, 34 thus, it is a naturally occurring, novel SNP candidate. Nucleotides within E6 DNA at positions 415 & 417 are within the HPV-45-E6* splice acceptor located at nucleotide 413 19, 30, 32 and may influence E6*I expression. Our current study, however, did not detect any nucleotide change(s) within or around the HPV-45- E6* splice donor site located at nucleotide 230. 30, 36 High-risk HPV E6 and E7 HPV oncogenes are transcribed by one promoter into a polycistronic pre-mRNA. The unspliced E6/E7 transcript encodes full-length E6 (E6fl) protein, while the E7 protein is derived from splice variants (spliced E6* variants). Human papillomavirus type-18 expresses the E6 fl and E6*I splice transcripts, and E6*I has been observed to correlate with reduced cellular proliferation of HPV-18-positive cancer cells. 37 Furthermore, HPV E6*I transcripts were observed to have anti-tumourigenic effects, including interfering with E6-mediated degradation of p53 and increasing cellular growth arrest. 32

The characterised E6/E7 SNPs of lineages/sublineages of HPV-18 (and/or HPV-45) could be linked to other polymorphisms occurring at other genome regions, but whole-genome sequencing for the complete classification of the variants was outside the scope of this study. This study was also limited by sample size. Thus, future studies in Ghana should consider expanding the number of samples to confirm the distribution of HPV-18 and HPV-45 lineage and sublineage variants in the country, ideally in individuals with and without disease, given the disease-enriched nature of this cohort. Additionally, though samples utilised in this study were positive for HPV L1 DNA and human β-globin, E6/E7 PCR product was not detected in five out of 33 samples (L1-HPV-18+) and nine out of 20 samples (L1-HPV-45+).

Thus, a nested PCR could have further minimised the number of samples without E6/E7 PCR product to rule out HPV-18 or HPV-45 low copy number/load issues. Other factors that might have impacted the E6/E7 PCR analysis could have been differences in detection assay (L1 versus E6/E7 testing), other HPV types with higher DNA concentration, and other HPV-18 (or HPV-45) variants. During the PCR, primers were designed to amplify HPV-18 or HPV-45 E6/E7 DNA (that is 896 b or 899 b, respectively) in one block using a 2-minute extension time, which is suitable for amplifying DNA up to 2 kb in length, thus considering PCR primers targeting E6 or E7 DNA separately in such instance may not change our results significantly.

In this report, HPV-18 and HPV-45 E6/E7 DNA variant lineages and sublineages were determined among a cohort of Ghanaian study participants, including over 70% confirmed cervical cancer cases. Thus, respective typespecific DNA variants in this report are likely to be clinically relevant and can inform future epidemiological studies. Our delineated sequences can contribute baseline data, vital for comparing HPV-18 or HPV-45 E6/E7 DNA variants after nationwide prophylactic HPV vaccination. The mapped sequences can be important for assessing E6/E7 primers for HPV-18 or HPV-45 testing in Ghana and in the African continent, where a higher burden of HPV-18 &HPV-45 genotypes has been reported compared to other parts of the globe. Our characterised type-specific HPV-18 or HPV-45 E6/E7 DNA sequences can inform studies evaluating HPV-18 (HPV-45) E6/E7 testing assays and strategies for prevention, treatment (therapeutic vaccines/antivirals) and management of cervical cancer cases in Ghana and Africa. Factors mediating carcinogenesis in a minority of individuals can be due to intrinsic viral and/or related risk factors.^38^ including HPV types and type-specific variants of HPV-18 and HPV-45. However, type-specific variants have different spread among discrete human populations. Though closely related, some have been reported with disparate carcinogenicity that may have diagnostic, treatment/management, and vaccine implications.^11^, ^21^, ^39^-^41^ and thus, further studies are needed. For example, for HPV-18, differential carcinogenicity among sublineages of lineage-A was observed in Asia, but no significant association of respective lineage variants with CxCa risk or histology types 14.

## Conclusion

This study is the first in Ghana to identify E6/E7 SNPs of HPV-18 and HPV-45 lineage/sublineage variants in cervicovaginal samples largely associated with high-grade disease and confirmed a dominance of HPV-45-sublineage-A1 and HPV-18-lineage-B together with a lower occurrence of the Africa-specific HPV-18-lineage-C.

This study will inform future studies on HPV-18 and HPV-45 E6/E7 detection/testing assay evaluation, mapping countrywide type-specific HPV variant spread, and when assessing the impact of respective variants on CxCa epidemiology.

Additionally, since population-based prophylactic vaccination against HPV infection is yet to be implemented in most developing countries, our identified E6/E7 DNA sequence variations in HPV-18 and HPV-45 could give useful information for E6 and/or E7-based therapeutic drug/vaccine formulation(s) against respective infection(s)/related diseases, which can help reduce the burden HPV infection/associated cancers, especially in Africa.
